# The development of a 4D treatment planning methodology to simulate the tracking of central lung tumors in an MRI‐linac

**DOI:** 10.1002/acm2.12233

**Published:** 2017-12-01

**Authors:** Shahad M. Al‐Ward, Anthony Kim, Claire McCann, Mark Ruschin, Patrick Cheung, Arjun Sahgal, Brian M. Keller

**Affiliations:** ^1^ Sunnybrook Health Sciences Centre Odette Cancer Centre Toronto ON Canada; ^2^ Department of Radiation Oncology University of Toronto Toronto ON Canada

**Keywords:** 4D treatment planning, central lung tumors, monaco TPS, MRI linac, SABR lung

## Abstract

**Purpose:**

Targeting and tracking of central lung tumors may be feasible on the Elekta MRI‐linac (MRL) due to the soft‐tissue visualization capabilities of MRI. The purpose of this work is to develop a novel treatment planning methodology to simulate tracking of central lung tumors with the MRL and to quantify the benefits in OAR sparing compared with the ITV approach.

**Methods:**

Full 4D‐CT datasets for five central lung cancer patients were selected to simulate the condition of having 4D‐pseudo‐CTs derived from 4D‐MRI data available on the MRL with real‐time tracking capabilities. We used the MRL treatment planning system to generate two plans: (a) with a set of MLC‐defined apertures around the target at each phase of the breathing (“4D‐MRL” method); (b) with a fixed set of fields encompassing the maximum inhale and exhale of the breathing cycle (“ITV” method). For both plans, dose accumulation was performed onto a reference phase. To further study the potential benefits of a 4D‐MRL method, the results were stratified by tumor motion amplitude, OAR‐to‐tumor proximity, and the relative OAR motion (ROM).

**Results:**

With the 4D‐MRL method, the reduction in mean doses was up to 3.0 Gy and 1.9 Gy for the heart and the lung. Moreover, the lung's V12.5 Gy was spared by a maximum of 300 cc. Maximum doses to serial organs were reduced by up to 6.1 Gy, 1.5 Gy, and 9.0 Gy for the esophagus, spinal cord, and the trachea, respectively. OAR dose reduction with our method depended on the tumor motion amplitude and the ROM. Some OARs with large ROMs and in close proximity to the tumor benefited from tracking despite small tumor amplitudes.

**Conclusions:**

We developed a novel 4D tracking methodology for the MRL for central lung tumors and quantified the potential dosimetric benefits compared with our current ITV approach.

## 1 INTRODUCTION

Respiratory and organ motion can have a substantial effect on treatment delivery in lung cancer radiation therapy. The main effect of respiratory motion on treatment delivery is dose blurring, defined as the reduced dose conformity due to the widening of the penumbra.^1^ In our clinic, we use the internal target volume (ITV) approach to manage respiratory motion as it captures the tumor at the full extent of the breathing motion. This approach, however, is overly conservative as the large beam apertures that are required to cover the tumor also irradiate the surrounding normal tissue and that limits the potential for dose escalation.

Common motion management strategies include gating and tumor tracking. With gating, the radiation beam is turned on only during a specific portion of the respiratory cycle. Gated radiation therapy, however, gives prolonged treatment times when compared with tumor tracking but does have the advantage of a more simplified approach.^2^ Tracking aims to mitigate the motion at the delivery stage as opposed to the planning stage as in the ITV technique. In traditional tumor tracking techniques, the radiation beam is synchronized with the tumor motion trajectory by using dynamic multi‐leaf collimators (dMLCs).^3^ This is achievable by means of an internal fiducial marker.^4^ However, these fiducials are invasive, and there is always a possibility for fiducial marker migration^5^ during the full course of the treatment, which may lead to inaccurate tumor tracking. Overall, the goal of tumor tracking is to allow for beam aperture reduction^6^ in order to improve OAR (organs at risk) sparing and/or to escalate the prescription dose when using reduced margins.^7^, ^8^ There are multiple reports that associate dose escalation with improved local regional control (LRC), which in turn improves the overall survival rates of cancer patients.^8^, ^9^, ^10^

The Elekta MRI‐linac or MRL (*Elekta AB, Stockholm, Sweden*) is positioned to provide a real‐time tracking functionality without the need of internal fiducials. Fiducials are not required due to the improved soft‐tissue contrast of MRI.^11^ Imaging during the treatment also allows us to monitor the treatment in real time. This system utilizes a closed‐bore 1.5 T MRI scanner with a linear accelerator rotating about its circumference. Other MRL systems include the commercial MRIdian^®^ from ViewRay^®^,^12^ the Cross Cancer Centre MRI‐linac prototype in Edmonton,^13^ and the Australian MRI‐linac under development in Sydney.^14^

In the near future, our cancer center will be installing the Elekta MRL. In anticipation of this installation, we developed a novel 4D treatment planning methodology for the MRL, which we then tested by demonstrating how tracking could possibly improve on OAR sparing for central lung tumors treated using stereotactic ablative radiotherapy (SABR) as compared with our current ITV approach. Four‐dimensional treatment planning is the incorporation of patient‐specific breathing patterns in the planning stage, in order to account for the dosimetric effects of respiratory motion.^15^, ^16^, ^17^ A few studies have developed methods for 4D treatment planning in IMRT^18^, ^19^, ^20^ and also a few for VMAT planning.^21^, ^22^ For example, a recent publication by Suh et al^23^ has demonstrated the development of a robust 4D optimization for lung cancer patients. It was also demonstrated in this work that 4D treatment planning could be used for offline inter‐fractional re‐planning and for online delivery.^23^ In terms of OAR sparing, the results from Trofimov et al^15^ show that conventional 3D planning approaches deliver more dose to nearby OARs than 4D treatment plans. In the literature, most 4D treatment planning methodologies have been developed using 4DCTs, and only a few papers discuss 4D methods for MRI.^24^, ^25^

**Figure 1:**
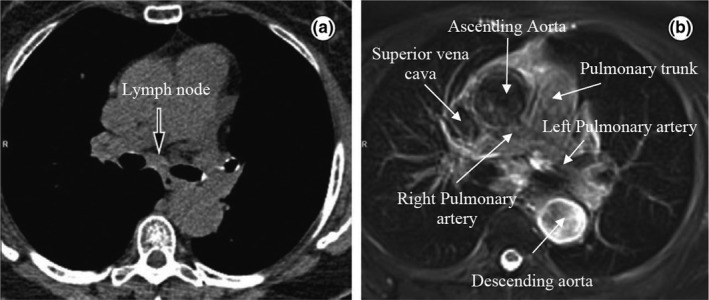
Axial slices of the mediastinum showing (a) a CT image and the corresponding (b) T_2_ weighted MRI image with improved soft‐tissue contrast. Some structures (the great vessels) are highlighted in the MRI image. Reprinted from Hochhegger et al.^26^

We chose central lung tumors because of the potential of MRI to discern the target from the surrounding critical organs, as exemplified in Fig. 1. This is particularly important in the context of stereotactic ablative treatments where high doses are delivered in a few fractions. Most often, dose prescriptions in this region need to be reduced, and so even small improvements in the visualization of the target and OARs followed by improvements in motion management could have a clinical impact. The present work details the development of our simulated tumor tracking method using the MRI‐linac beam model (along with a virtual couch shift) within the Monaco treatment planning system that will be used with the MRL. This work also quantifies the potential benefits of tumor tracking in terms of OAR sparing for central lung tumors.

## 2 METHODS AND MATERIALS

### 2.A Simulated tumor tracking for the MRI‐linac

We developed a 4D treatment planning methodology (4DMRL) to simulate real‐time tumor tracking in an MRL system using the Monaco treatment planning system (research version 5.19.00). The newly developed dose calculation algorithm, GPUMCD, is a Monte Carlo‐based dose calculation algorithm capable of modeling dose in the presence of a magnetic field for the 7 MV nominal energy MRL beam.^27^ The electron return effect is modeled in this algorithm, and this effect is incorporated within the results. This algorithm was recently benchmarked against a widely used Monte Carlo algorithm GEANT4.^28^

**Figure 2:**
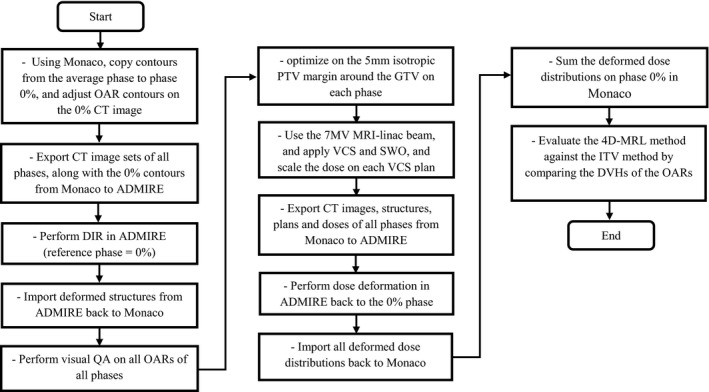
Developed workflow of the 4D‐MRL planning procedure created in the Monaco TPS.

The details of the 4DMRL method are outlined in Fig. 2. In brief, 4DCT data from five NSCLC (non–small‐cell lung cancer) patients were used to develop and test our method. These patients were prescribed with SABR (Stereotactic Ablative Radiation Therapy). The contours from the average intensity CT image were copied to the maximum inhale phase (0% phase) of the 4DCT which we designated as the reference phase. The OAR contours were manually adjusted to match the anatomy of the structures on the inhale phase. The contours of the GTV (Gross Tumor Volume) at each phase were reviewed by a staff radiation oncologist. The inhale phase contours were then propagated to all of the other breathing phases using the research version of ADMIRE (Advanced Medical Imaging Registration Engine, Elekta AB, Stockholm, Sweden), a deformable image registration (DIR) software. We used a cascade DIR algorithm (i.e., deformable registration is cascaded from one phase to the next). The resulting automated contours on all of the phases were imported into the Monaco planning system and visually verified.

When delivering conventional intensity modulated radiation therapy (IMRT) treatments, couch translations and rotations may be exploited to account for discrepancies in patient‐specific setup errors between the CT simulation and the cone beam CT (CBCT). In the MRI‐linac system, however, couch translations and rotations are not physically achievable, rather appropriate adjustments are made using software that is able to shift an aperture to a new location (a so‐called virtual couch shift). Therefore, to simulate tumor tracking, we applied a virtual couch shift (VCS),^29^, ^30^ which shifts the apertures on each phase to match that of the reference phase while maintaining a constant isocenter location. The VCS maintains all patient‐specific pretreatment dose constraints and delivers a clinically comparable dose distribution at a new position.^29^ Each VCS plan was optimized using segment weight optimization (SWO).

A 5‐mm isotropic margin around the GTV called the planning target volume (PTV) was targeted in each VCS plan using the same dose constraints as the ITV method. The optimized dose on each phase was given a certain weight based on the pattern presented in a pre‐acquired patient‐specific breathing trace. All plans used the IMRT technique, and they were calculated using a 0.25‐cm dose grid resolution and a statistical uncertainty in Monaco of 1% per calculation (i.e., 1% in the central region of the target volume).

After applying the VCS and the dose weighting, the dose was normalized such that the target's V99% was 100%, as was the case in the ITV method. All image sets, contours, plans, and doses were exported to ADMIRE for dose deformation. ADMIRE mapped the doses from each phase to the inhale phase. The deformed doses were imported back to Monaco and accumulated on the inhale phase resulting in a single summation plan (the 4D‐MRL plan).

The dose on our 4D‐MRL plan was accumulated on the 0% image using deformed doses from all CT image sets. However, our ITV plan was only calculated on the 0% CT image set. A reasonable comparison with the 4D‐MRL method would be to create an ITV plan with an algorithm similar to the 4D‐MRL method. We called this ITV plan the accumulated ITV plan (ITV_acm_). In other words, this ITV plan was performed by first optimizing a plan on the PTV using the 0% phase data set. Then, a copy of this plan was created and appropriately weighted for each phase. Finally, the doses from each plan were deformed, propagated (using ADMIRE), and accumulated back on the 0% phase. Our conventional ITV plan was compared with the ITV_acm_ plan for one patient, and there were no notable differences in the dose statistics between the two plans.

To test our developed method, we evaluated OAR sparing using dose volume histograms (DVHs) and dose parameters from AAPM TG‐101.^31^ We investigated the advantages of using the MRL tracking method (which we plan to use in the future) over our current practice in the clinic which uses the ITV method of motion management in a nonmagnetic field on a standard 6 MV Elekta Infinity linac. The DVHs and dose statistics from the 4D‐MRL plans were compared with those of the ITV plans.

#### 2.A.1 Patient selection

**Figure 3:**
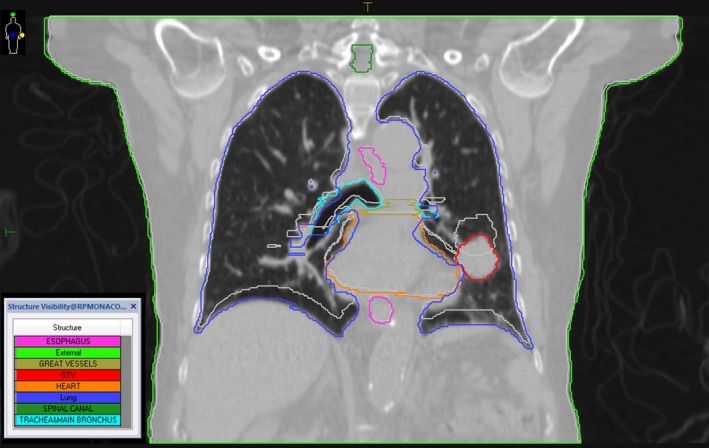
GTV and OAR contours on a coronal section for patient 1 at phase 0% (the colored contours) and phase 50% (the light gray contours). The CT image shown is that of phase 0%.

Five SABR NSCLC patients with central tumors were chosen. We selected centrally located lung tumors for this study due to the proximity of these tumors to vital organs such as the heart, the esophagus, the trachea, main bronchus, and great vessels of the heart and the strong potential for MRI to better delineate the tumor and normal organ boundaries in this region. All data sets used in this study were 4DCTs from clinical lung SABR patients treated using our ITV method of motion management. 4DCTs were acquired using a Philips Big Bore scanner (Cleveland, OH, USA) with a slice thickness of 3 mm reconstructed every 1.5 mm and using 140 kVp. Figure 3 shows the GTV and OAR contours on a coronal section for one of the five patients at phase 0% and 50%. Patient data (Table 1) included eight phases of amplitude binned 4DCT images, contours of the ITV, GTV, and OARs on a reference image set, and patient‐specific breathing traces. The data were retrospectively collected and analyzed.

**Table 1:** Five SABR lung cancer patients with central lung tumors were chosen. The motion amplitude was measured by taking the vector differences between the centroids of the GTV0 and the GTV50 (End‐exhale)

Patient	Prescription/no. fractions	Motion amplitude (cm)	GTV volume (cm^3^)	Tumor location
1	52 Gy/4	1.74	15.19	Central right
2	50 Gy/5	0.12	6.09	Central left
3	50 Gy/5	0.25	21.33	Central right
4	50 Gy/5	0.86	1.29	Central right
5	50 Gy/5	0.18	12.01	Central right

#### 2.A.2 Patient‐specific breathing traces

**Figure 4:**
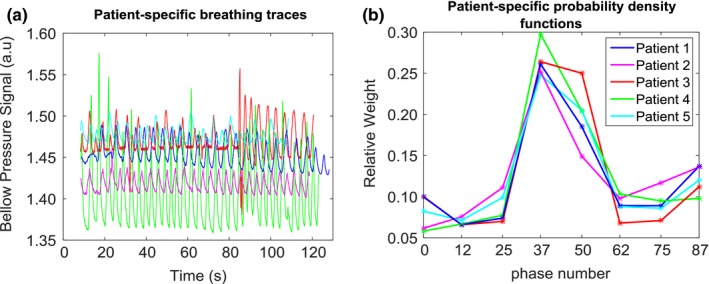
(a) Patient‐specific breathing traces plotted as bellow pressure signal (a.u.) vs. time (s). These traces were acquired by means of rubber air bellows. (b) Patient‐specific breathing traces are binned to provide patient‐specific PDFs.

Patient‐specific breathing traces were acquired [Fig. 4(a)] using abdominal bellows during 4DCT image acquisition. Since the 4DCT images were amplitude binned, we needed to define the average fraction of time each patient spent in each breathing phase. To accomplish this, a custom MATLAB code was created. First, a moving average filter of size 50 was used (one output data point for every 50 input data points) to reduce noise in the breathing signal, and then the breathing peaks were identified to determine the length of each period. The breathing trace was binned creating a probability density function (PDF)^32^ for each period. For each patient, the PDFs for all periods were averaged and this average was used to weight the dose on each phase [Fig. 4(b)].

#### 2.A.3 Relative organ motion

We also investigated the effects of (a) relative OAR motion and (b) the proximity of the OAR to the target on dose sparing. ROMs for different OARs were calculated using the following formula:

(1) $$ROM\left(OAR\right)=|d_{\mathrm{exh}}-d_{\mathrm{inh}}|$$

where *d*_*inh*_ is the minimum distance between the GTV at phase 0% and the intended OAR, and *d*_*exh*_ is the minimum distance between the GTV at phase 50% and the OAR. We also used *d*_*inh*_ as the measure for the proximity of the OAR to the target.

## 3 RESULTS

### 3.A Clinical impact of tracking

**Figure 5:**
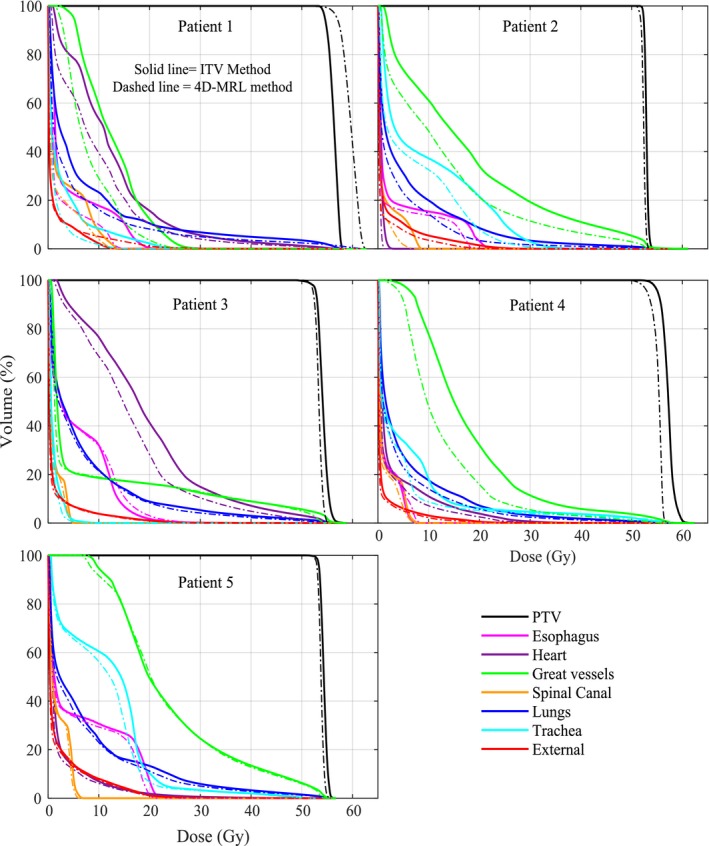
Dose volume histograms from the two treatment planning methods for the five patients using the ITV method and the 4D‐MRL method.

**Figure 6:**
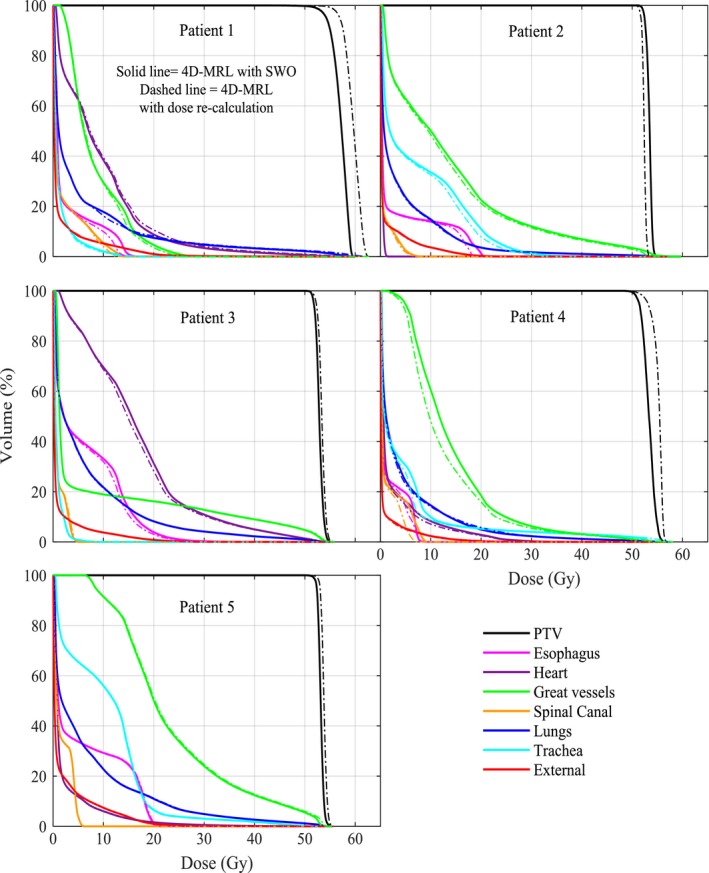
Dose volume histograms from the 4D‐MRL method using SWO on each phase versus a simple dose recalculation on each phase.

The benefits of using an MRL tracking system are summarized in Fig. 5 and Table 2. The reduction in mean doses to parallel organs ranged from 0.3 Gy to 3.0 Gy for the heart and 0.6 Gy to 1.9 Gy for the lung. The absolute difference in maximum doses to serial organs ranged from 0.6 Gy to 6.1 Gy for the esophagus, from 0.3 Gy to 1.5 Gy for the spinal canal, and from 0.1 Gy to 9.1 Gy for the trachea and main bronchus. This all considers the irradiation within an orthogonal magnetic field. To verify that the dose reduction in the 4D‐MRL method was in fact due to tracking and not merely due to the re‐optimization process, we re‐performed the 4D‐MRL method following the same steps as in Fig. 2, except that we did not perform segment weight optimization (SWO) on each phase. Instead, we simply re‐calculated the dose on each VCS plan. Figure 6 indicates that there were no noticeable differences in the 4D‐MRL plan DVHs when simply tracking the target versus optimizing on each of the phases.

**Table 2:** Clinically relevant parameters for each patient using the ITV method and the 4D‐MRL method. The SABR limits were referenced from AAPM TG‐101.^31^ Bold values indicate a notable reduction in OAR doses

OAR	Parameter	SABR limits	End point (>grade 3)	Patient 1 Tumor amplitude =1.74 cm		Patient 2 Tumor amplitude = 0.12 cm		Patient 3 Tumor amplitude = 0.25 cm		Patient 4 Tumor amplitude = 0.86 cm		Patient 5 Tumor amplitude = 0.18 cm	
				ITV	4D‐MRL	ITV	4D‐MRL	ITV	4D‐MRL	ITV	4D‐MRL	ITV	4D‐MRL
ESO	V19.5 Gy (cc)	<5 cc	Stenosis/fistula	0.2	0.0	1.7	0.0	0.8	1.2	0.0	0.0	3.6	1.1
	Max dose (Gy)	<35 Gy		**21.3**	**15.2**	**23.5**	**19.0**	39.5	40.1	8.1	8.8	23.2	22.0
	*d*_*inh*_ (cm)			3.95		3.75		1.1		3.05		2.2	
	ROM (cm)			0.35		0.35		0.00		0.45		0.30	
HRT	V32 Gy (cc)	<15 cc	Pericarditis	**32.9**	**23.5**	0.0	0.0	**79.2**	**54.6**	**2.4**	**0.4**	3.4	3.3
	Max dose (Gy)	<38 Gy		**60.3**	**57.1**	3.6	2.0	**57.5**	**54.7**	**44.5**	**40.4**	53.5	52.8
	Mean dose (Gy)			12.4	9.7	0.6	0.3	19.1	16.1	3.3	2.5	2.7	2.4
	*d*_*inh*_ (cm)			−0.58		2.67		−0.3		1.48		0.5	
	ROM (cm)			0.58		0.53		0.1		0.52		0.00	
LNG	V12.5 Gy (cc)	<1500 cc	Pneumonitis	**530**	**394**	**846**	**539**	580	580	547	426	518	503
	V13.5 Gy (cc)	<1000 cc		**468**	**369**	**785**	**469**	530	528	507	392	474	465
	Mean dose (Gy)			7.5	5.8	5.9	4.0	7.1	6.5	5.5	4.5	7.7	7.0
SC	V23 Gy (cc)	<0.35 cc	Myelitis	0.0	0.0	0.0	0.0	0.0	0.0	0.0	0.0	0.0	0.0
	Max dose (Gy)	<30 Gy		14.1	12.6	8.8	8.2	5.6	5.3	8.1	7.5	6.8	6.5
	*d*_*inh*_ (cm)			7.5		7.5		4.0		6.2		4.3	
	ROM (cm)			0.30		0.00		0.00		0.0		0.00	
GRT	V47 (Gy)	<10 cc	Aneurysm	0.0	0.0	**17.9**	**10.2**	10.7	10.0	**4.6**	**1.8**	16.2	15.2
	Max dose (Gy)	<53 Gy		**31.9**	**28.0**	**60.7**	**57.4**	**58.6**	**55.6**	58.1	56.3	55.9	55.1
	*d*_*inh*_ (cm)			1.22		−0.29		0.05		1.17		0.0	
	ROM (cm)			1.28		0.29		0.05		1.23		0.0	
TRA	V16.5 (Gy)	<4 cc	Stenosis/fistula	1.5	0.0	**17.9**	**10.3**	0.0	0.0	2.8	2.2	**12.9**	**7.3**
	Max dose (Gy)	<40 Gy		**26.3**	**17.3**	**45.3**	**42.2**	9.4	7.9	57.7	57.8	51.5	51.7
	*d*_*inh*_ (cm)			1.02		0.92		1.85		0.03		−0.1	
	ROM (cm)			0.78		0.38		0.05		0.07		0.3	

ESO, esophagus; HRT, heart; LNG, lungs; SC, spinal canal; GRT, the great vessels.

Dose reduction did not only depend on motion amplitude. Both ROM and the OAR proximity to the target had an effect on dose sparing. For example, although patient 2 had small motion amplitude (0.12 cm), the overall reduction in doses to the OARs did have an impact. This is attributed to the relative OAR motion (ROM) (Table 2). Patients 3 and 5 did not have a notable OAR dose reduction when using the 4D‐MRL method as these two patients had a small tumor motion (0.25 cm and 0.18 cm, respectively) and a small ROM. Patient 4 had a notable reduction in the maximum dose to the heart (Table 2) and to the total dose to the great vessels (Fig. 5). The great vessels in patient 4 had a *d*_*inh*_ of 1.17 cm and a large ROM value of 1.37 cm.

**Figure 7:**
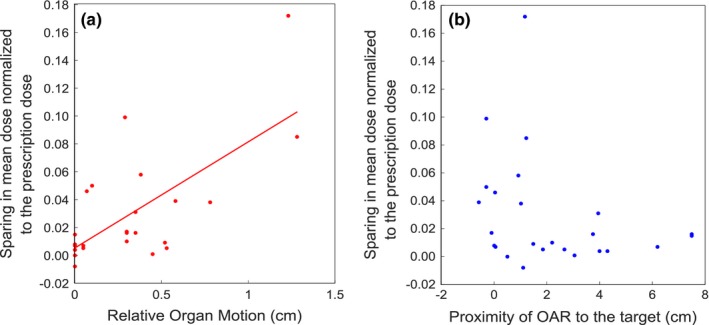
Scatter plots illustrating the sparing in OAR mean dose as a function of relative organ motion (ROM) or proximity of OAR to the target, for all OARs. The solid line in (a) represents a linear curve fit to the data points. In (b), as the target to OAR distance increases beyond about 1.5 cm, there is little to no effect on dose sparing. For OARs in (b) that move into the target volume, the *x*‐axis data points are shown as less than zero.

To further quantify dose sparing as a function of the ROM, we performed a linear regression analysis using MATLAB (Fig. 7). Dose sparing was calculated by taking the differences between the mean doses of the OARs when planned using the ITV method versus the 4D‐MRL method, then normalizing these differences to the prescription dose. The results of this test gave a *P*‐value of 0.0001 for the relationship between dose sparing and ROM. This indicated that dose sparing became more significant as ROM increased. Figure 7 also shows a plot of dose sparing as a function of the proximity of the OAR to the target. Interestingly, for OARs that are close to the target (within about 1.5 cm), dose sparing is evident in a number of cases. However, as the OAR to target distance increases beyond about 2 cm, there is little to no effect. In general, tumor motion for all patients was primarily in the cranial–caudal direction, with the exception of patient 4 for whom the lateral–medial component of the tumor motion was more dominant than the cranial–caudal one.

## 4 DISCUSSION

We developed a method to simulate tumor tracking in a magnetic field using the Monaco treatment planning system and the ADMIRE deformable image registration software both of which will be used in Elekta's commercial MRI‐Linac system (MRL). This method was then used to evaluate the benefits of tumor tracking in the MRL compared with what we currently do, which is the ITV method of motion management on a conventional Elekta Infinity linac. There is a motivation to improve on the conventional ITV approach of motion management, particularly in cases where the organs‐at‐risk are in close proximity to the target. This combined with advances in real‐time MRI imaging through an MRI‐linac solution provides a potential avenue to dose escalate the tumor and/or spare OARs.

We chose central lung tumors because of the complex interplay of the target and OAR motions in this region, as well as the capability of MRI to visualize soft tissue. Although this manuscript has focused on central lung tumors, this methodology would certainly apply to a number of other anatomical locations. For example, abdominal tumors, such as liver and pancreas, would benefit from the soft‐tissue contrast afforded by MRI. Additionally, abdominal tumors located near the diaphragm can be particularly susceptible to large motions, and as such, a motion management strategy is required. The number of studies evaluating the dosimetric effects of motion in a magnetic field for commercial systems is limited. A recent study by Menten et al investigated the impact of an orthogonal magnetic field on tumor tracking in lung SABR, demonstrating that the optimization process is capable of mitigating the electron return effect at tissue–lung interfaces.^33^ However, the Menten et al study did not investigate the effects of OAR sparing for central lung tumors and the clinical impact.

Target motion was the main factor affecting OAR sparing and this was evident from Fig. 5, where patient 1 had the largest target motion and quite notable OAR sparing. That being said, regardless of the target motion, the results have shown that OARs benefited from MRL tracking under two conditions, (a) when the ROM was ≥0.3 cm and (b) when the OAR was in close proximity to the target. For example, patient 2 had a small target motion (on the order of millimeters) with ROM values greater than 0.3 cm for the esophagus and the trachea, and there was a considerable reduction to the maximum doses to these OARs. Patient 3, on the other hand, had ROMs of essentially zero for the esophagus, spinal cord, and trachea, and the dose reduction to these organs was barely notable. Other organs like the heart in patient 1 had a ROM of 0.58 cm, but there was no notable sparing to the heart, most likely due to the large distance of the heart from the target (3.23 cm) in this case. This is also illustrated in Fig. 7.

In terms of the lung itself, several factors may affect lung sparing such as large volume changes during respiration and the resultant changes in tissue density. However, this study involved patients with central lung tumors, meaning that most of the tumors were fairly attached to soft tissue rather than situated inside the lung. The results in Fig. 5 show that only patients 1 and 2 had a considerable lung sparing. A close examination of our CT data sets revealed that the tumor ranges of motion in patients 1 and 2 involved more lung tissue than the other patients so the benefits were most pronounced for these two patients.

Radiation‐induced biological effects were assessed in this study based on the parameters found in the AAPM TG‐101 report.^31^ The reported parameters indicated biological end points of grade 3 or higher, which ranged from the occurrence of a severe adverse event to death related to an adverse effect.^34^ This report, however, does not indicate the probability of occurrence of each biological end point. These probabilities are rather found in the QUANTEC report,^35^ but these are based on the conventional 2 Gy per fraction regimen. We did some calculations of normal tissue complication probability (NTCP) using the Lyman–Kutcher–Burman (LKB) model,^36^ and the values were low due to the insensitivity of this model to low equivalent uniform dose (EUD) values. The EUD values were low since we strove to create treatment plans that met our clinical criteria for lung SABR, so in the end, our NTCP values were fairly insensitive to changes in our DVH curves and were not relevant to report.

One of the limitations of this study was that we simulated tracking based on a fixed breathing pattern acquired prior to the planning process. The patient breathing may vary in amplitude, period, and/or baseline drifts. Typical breathing periods published in the literature last from 2.7 to 6.6 s with an average of 5 s and the typical tumor amplitudes range from 6 to 18 mm.^37^ Due to these uncertainties, the robustness of the 4D‐MRL method is not definitively ascertained, rather, our results represent a best case scenario for the tracking of a moving target. We assumed an ideal scenario in which there were no deviations from this pattern, although current research in the field is evaluating the robustness of dose distributions to small motion deviations in a magnetic field.^38^ We also assumed direct tracking of the tumor with high fidelity and no latency in the aperture tracking. A study by Cerviño et al^39^ investigated the accuracy of MRI‐guided tumor tracking in lung cancer radiotherapy using cine‐MRI and two algorithms: (a) an artificial neural network (ANN) algorithm and (b) a template matching algorithm (TM). The results of this study showed that TM yields an average prediction error of 0.6 mm and an error at the 95% confidence level of 1.0 mm for both regular and irregular breathing. These results are promising, as they show the potential for accurate tumor tracking using cine‐MRI. The latency associated with the current Elekta MRL system using cine‐MRI is of the order of 100–200 ms. It is expected that imposing practical constraints such as latency will cause dose to be blurred (relative to planned) and affect the overall accuracy of delivered dose, compared to the theoretical benefit shown in our manuscript. Although outside the scope of the present study to investigate these effects, Roland et al^40^ have investigated this effect on a phantom, using a different linac tracking system (TracBeam, Initia Medical Technologies, Petah Tikva, Israel). This system had a latency of 172 ms, and a film gamma analysis comparing measurements with the treatment plan suggested that 98% of pixels passed the 3%/3 mm criteria when accounting for latency using a convolution approach in the planning system. If latency was not accounted for, the gamma pass rate was reduced to 93%, suggesting that accounting for latency in the planning system improved the pass rate by 5%.

**Figure 8:**
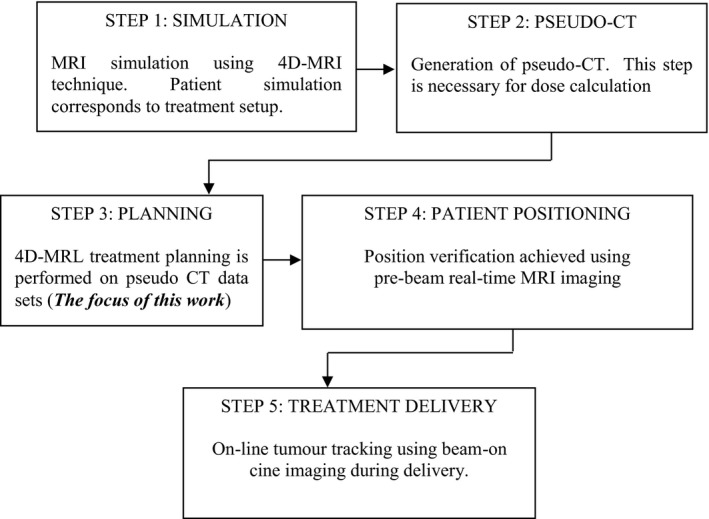
MR‐Only workflow for tumor tracking in the MRL.

Currently, CT imaging is part of the radiation therapy workflow. With the installation of the MRL, it would be advantageous to move away from a CT workflow to an MR‐only workflow. Köhler et al described a solution for an MR‐only based planning workflow for the Philips Ingenia scanner using Magnetic Resonance for Calculating ATenuation (MRCAT) for external beam radiation therapy for prostate and cervical cancer.^41^ MR images do not provide electron densities required for dose calculation. However, CT‐like density maps are generated by classifying the contents of the MR image into different tissue types, and then each voxel is assigned a “pseudo‐HU value.” Figure 8 shows our suggested workflow for tumor tracking in the MRI‐linac, which is similar to the workflow described by Köhler et al^41^ Also relevant to our suggested workflow is a recently published manuscript by Deng et al.^42^ This group proposed a 4D‐MRI technique that characterizes respiratory motion in human subjects. This method compared well against real‐time 2D‐MRI using phantoms and patients. The acquisition of the 4D‐MRI is the first step in the proposed workflow, just prior to the conversion to 4D‐CT for treatment planning.^42^

MRI of the lung is challenging in radiotherapy as a result of (a) the low proton density in the lung and (b) the presence of tissue–air interfaces, which generates susceptibility artifacts and ultimately causes signal loss.^26^ Our study, however, focused on central lung tumors where the proton density is higher than the rest of the lung. Furthermore, a study by Biedere et al has shown that MRI is beneficial in patients with pathological conditions, as the proton density in the lung is increased.^43^ In addition, the treatment of lung tumors using MRI for planning and real‐time guidance is at its infancy and will develop over time as MRI‐linac configurations become clinical. Two real‐time imaging methods are possible using fast echo planar imaging^44^ or in‐room cine‐MRI guidance imaging.^45^, ^46^

This study can be regarded as an initial investigation of the potential benefits of using an MRI‐linac tracking system for centrally located lung tumors affected by breathing induced motion. Indeed, more work needs to be done in terms of validating the applicability of our method with respect to treatment delivery. The dose distribution from our 4D‐MRL plans may not necessarily be comparable to the delivered dose of these plans. Therefore, the assumption that the 4D‐MRL plan is superior to the ITV plan needs to be validated in the future. Interestingly, Wang et al have generated lung pseudo‐CTs from MRI images and compared the dosimetry of the pseudo CTs to the standard CTs. Their Gamma analysis has shown a pass rate of 99.3 ± 1.1% for the 2%/2 mm acceptance criteria for their plans.^47^ It is also worth noting that we have not decoupled the effects of the magnetic field on the treatment beam for the 4D‐MRL study arm, although the effects of the magnetic field are inherent within the results. Moreover, the MRL beam model itself has undergone some preliminary experimental validation both by Elekta and academic centers, and initial results suggest good agreement between the Monaco model and experimental measurements, giving more credence to the results presented in this study.

One notable difference between the 6 MV Infinity beam and the 7 MV MRL beam is the relatively large SAD (source to axis distance) of the MRL (143.5 cm) when compared to that of the Infinity (100 cm) resulting in a projected leaf width for the MRL beam of 7.2 mm compared to 5 mm for the Agility. That being said, the results shown here demonstrate that this difference does not seem to have a detrimental effect to cancel out the positive effects of tumor tracking.

Last, it is worth commenting on the PTV margin used. There needs to be a margin study for a given tumor site designed to calculate the appropriate PTV margin given the equipment used. This accumulation of experimental evidence can generate an appropriate PTV margin for lung tumors treated in an Elekta MRI‐linac. We decided on a 5 mm PTV margin as a reasonable starting point as this is what we currently use for our SABR lung patients. Using this margin, the 4D‐MRL tracking method reduced the irradiated volume by an average of 29% over all patients. In the future, the PTV margin may be smaller than 5 mm due to a more accurate treatment delivery and image guidance chain. The reduction of the PTV margin would lead to further reduction of the dose to nearby critical organs and to smaller irradiation fields that permit dose escalation.

## 5 CONCLUSIONS

This work investigated the benefits of tracking central lung tumors in an MRI‐linac. A 4D treatment planning method was developed in the presence of a magnetic field and OAR DVHs and TG‐101 parameters were compared against those obtained using our conventional ITV method. Four‐dimensional planning, in the presence of the magnetic field, caused target margin reduction which in turn caused a reduction in doses to the nearby critical organs. The degree of OAR reduction depended on the extent and direction of the target motion, the relative distance between the OAR and the target, and the relative target motion. There did not appear to be a deleterious effect in the tracking arm of the study due to the strong magnetic field. The results of this work have quantified the potential benefit of tumor tracking in the presence of the magnetic field when treating central NSCLC tumors.

## CONFLICT OF INTEREST

This project was made possible with the financial support of Elekta AB, Stockholm, Sweden.
